# Health equity: evidence synthesis and knowledge translation methods

**DOI:** 10.1186/2046-4053-2-43

**Published:** 2013-06-22

**Authors:** Vivian A Welch, Mark Petticrew, Jennifer O’Neill, Elizabeth Waters, Rebecca Armstrong, Zulfiqar A Bhutta, Damian Francis, Tracey Perez Koehlmoos, Elizabeth Kristjansson, Tomas Pantoja, Peter Tugwell

**Affiliations:** 1 Institute of Population Health, University of Ottawa, 1 Stewart Street, Ottawa, ON K1N6N5, Canada; 2 Department of Social and Environmental Health Research, London School of Hygiene and Tropical Medicine, London, UK; 3 Victorian Health Promotion Foundation, Carlton, Australia; 4 Division of Women and Child Health, Aga Khan University, Karachi, Pakistan; 5 Epidemiology Research Unit, University of the West Indies, Mona, Jamaica; 6 College of Health and Human Services, George Mason University, Fairfax, Virgina, USA; 7 Department of Family Medicine, Pontificia Universidad Catolica de Chile, Santiago, Chile; 8 Melbourne School of Population Health, The University of Melbourne, Melbourne, Australia; 9 Department of Medicine, University of Ottawa, Ottawa, Canada; 10 Epidemiology and Community Medicine, Faculty of Medicine, University of Ottawa, Ottawa, Canada; 11 Clinical Epidemiology Program, Ottawa Hospital Research Institute, Ottawa, Canada; 12 School of Psychology, University of Ottawa, Ottawa, Canada

**Keywords:** Health Equity, Evidence Synthesis, Knowledge Translation, Systematic Reviews

## Abstract

**Background:**

At the Rio Summit in 2011 on Social Determinants of Health, the global community recognized a pressing need to take action on reducing health inequities. This requires an improved evidence base on the effects of national and international policies on health inequities. Although systematic reviews are recognized as an important source for evidence-informed policy, they have been criticized for failing to assess effects on health equity.

**Methods:**

This article summarizes guidance on both conducting systematic reviews with a focus on health equity and on methods to translate their findings to different audiences. This guidance was developed based on a series of methodology meetings, previous guidance, a recently developed reporting guideline for equity-focused systematic reviews (PRISMA-Equity 2012) and a systematic review of methods to assess health equity in systematic reviews.

**Results:**

We make ten recommendations for conducting equity-focused systematic reviews; and five considerations for knowledge translation. Illustrative examples of equity-focused reviews are provided where these methods have been used.

**Conclusions:**

Implementation of the recommendations in this article is one step toward monitoring the impact of national and international policies and programs on health equity, as recommended by the 2011 World Conference on Social Determinants of Health.

## Background

The recommendations of the World Conference on Social Determinants of Health (Rio de Janeiro, 19–21 October 2011) recognized the pressing need to take action on reducing health inequities; one of its key recommendations was to assess the effects of national and international policies on health inequities [1]. Effects of interventions on health equity are also of paramount importance for health systems research and decision-makers [2,3]. The need for considering health equity is recognized for clinical health care and preventive interventions as well as place-based programs in disadvantaged areas or communities, and the social gradient in effects of population-based strategies to promote and maintain health [4-6]. In this era of fiscal restraint, there is a critical need for evidence about how to improve health equity in the most efficient way [7].

Systematic reviews are widely recognized as an efficient, reliable and comprehensive source of evidence for decision-making. Few systematic reviews have considered effects on health equity, even though research methods to assess effects on health equity in systematic reviews have been available and recently have been strengthened for use within natural policy experiments and systems approaches [3,8,9].

Several groups have documented methodological challenges when considering effects on equity in systematic reviews. For example, methods are needed to define the underlying theory and the mechanisms by which the intervention is expected to affect health equity [10]. Also, the search strategy may need to encompass a broader range of electronic and gray literature sources [8]. Methods to assess the influence of context and its relevance for discussion of applicability are needed.

Knowledge translation (KT) of the results on systematic reviews on equity is essential to ensure the results are utilized. KT is defined by the Canadian Institutes of Health Research as a ‘dynamic and iterative process that includes synthesis, dissemination, exchange and ethically-sound application of knowledge to improve health, provide more effective health services and products and strengthen the health care system’ [11]. Comprehensive KT is important to maximize the benefit from funding and conducting knowledge syntheses, both in terms of improving health of disadvantaged populations and for informing research priority setting exercises that consider prioritized effects on health equity. By clearly identifying disadvantaged populations, implementation of new policies can be targeted to those who most need them.

The purpose of this article is to provide guidance on how to conduct equity-focused systematic reviews consistent with the recommendations of PRISMA-E 2012 to facilitate the use of both guidance documents. This article also discusses challenges related to knowledge translation for equity-focused systematic reviews.

## Methods

We developed these recommendations based on methodology meetings held between 2005 and 2012 by the Campbell and Cochrane Equity Methods Group, methodological recommendations from the Cochrane Public Health Review Group [12], a Cochrane systematic review [13], methods study [14], the WHO Task Force on evidence-informed policies about health systems [2] and a consensus meeting held in Bellagio, Italy, in February 2012 with methodologists, funders, journal editors, clinicians and public health practitioners as part of the development of reporting guidelines for systematic reviews with a focus on health equity to extend the PRISMA (Preferred Reporting Items for Systematic Reviews and Meta-Analyses) statement (PRISMA-E 2012) [15].

### What is an equity-focused systematic review?

Health inequities are defined as differences in health outcomes that are avoidable, unfair and unjust [16]. Health inequities persist and are worsening for some conditions across population and individual characteristics both within and across countries. The Campbell and Cochrane Equity Methods Group and the Cochrane Public Health Group recommend the PROGRESS-Plus acronym to identify population and individual characteristics across which health inequities may exist. PROGRESS-Plus stands for place of residence, race/ethnicity/culture/language, occupation, gender/sex, religion, socioeconomic status and social capital, and “plus” captures other characteristics that may indicate a disadvantage, such as age and disability [17,18]. The use of an acronym such as PROGRESS-Plus helps explicitly and systematically consider health equity in the design of both primary studies and systematic reviews.

Systematic reviews with a major focus on health equity are those designed to:

(1) Assess effects of interventions in disadvantaged population(s) (such as school feeding for disadvantaged children) [19];

(2) Assess effects of interventions aimed at reducing social gradients across populations (e.g., interventions to reduce the social gradient in smoking) [20]; and/or

(3) Assess effects of interventions not aimed at reducing inequity but where it is important to understand the effects of the intervention on equity, either positively or negatively (e.g., an intervention targeted at the whole population but that may have effects on equity, such as the review on obesity prevention in children, which examined the effects of interventions across relevant PROGRESS-Plus factors) [21].

We have estimated that at least 20% of systematic reviews indexed in MEDLINE meet one or more of the above criteria [15]. We have assembled a selection of exemplar reviews that highlight one or more of the methodological challenges discussed in this article (Table 1). These reviews were identified by participants in the above meetings and by searching for systematic reviews in PubMed, the Cochrane Database of Systematic Reviews and the Campbell Library using the text words “equity” or “inequity”.

**Table 1:** Exemplar systematic reviews

**Systematic reviews with a major focus on health equity**	**Example exemplar review**	**How equity was considered**
(1) Assess effects of interventions in disadvantaged population(s)	School feeding for improving the physical and psychosocial health of disadvantaged students [19]	This review included only studies in which the intervention was target at ’predominantly disadvantaged’ children (e.g., living in a rural area or village, or an urban area and described as socioeconomically disadvantaged, from poor areas, if 30% of more of the children in the sample were underweight, or stunted or the average weight, height and body mass index (BMI) were low, or if the studies were implicitly or explicitly aimed at disadvantaged children (and indicators of disadvantage were provided) [19]
	Interventions to reduce the prevalence of female genital mutilation/cutting in African countries [22]	Female genital mutilation/cutting (FGM/C) is practiced mainly on young girls and has many potential adverse effects. In addition to ethnic, cultural and religious beliefs, there are socioeconomic beliefs that FGM/C is required for marriage or an economic necessity when women are dependent on men. This review examined intervention features and contextual factors that reduce the prevalence of FGM/C [22]
	What is the impact of microfinance on poor people? A systematic review of evidence from Sub-Saharan Africa [23]	This review aimed to determine the impact of microfinance interventions on incomes of the poor, on wider poverty/wealth of the poor and on non-financial outcomes, such as health and food security. The authors found that microfinance had inconclusive effects on savings and income but positive effects on health outcomes [23]
(2) Assess effects of interventions aimed at reducing social gradients across populations	Population tobacco control interventions and their effects on social inequalities in smoking: systematic review [20]	This systematic review applied an “equity lens” to population level interventions to reduce inequalities in smoking rates and extracted outcome, process and implementation data stratified by PROGRESS-Plus. Certain interventions, such as smoking restrictions in schools, restricting sales to minors and increasing the price of tobacco, are more effective in reducing smoking among lower-income adults and those with manual occupations. Other interventions had no effect on reducing social inequalities in smoking [20]
	Working for health? Evidence from systematic reviews on the effects of health and health inequalities of organizational changes on the psychosocial work environment [24]	The psychosocial work environment has a strong gradient that influences inequalities in health. This umbrella review examined the impacts of interventions on inequalities in health by socioeconomic status, age, gender and ethnicity and found that some organizational workplace interventions can reduce health inequalities in those who are employed, especially between men and women, and socioeconomic groups [24]
	Socioeconomic differences in lung cancer incidence: a systematic review and meta-analysis [25]	The socioeconomic gradient in lung cancer results from differences in exposures and risk factors, such as smoking, occupational/environmental exposure to inhaled carcinogens and air pollution. This meta-analysis found that lung cancer risk was highest among those in the lowest socioeconomic categories for education, occupation and income [25]
(3) Assess effects of interventions not aimed at reducing inequity but where it is important to understand the effects of the intervention on equity, either positively or negatively	Interventions for preventing obesity in children [21]	This review extracted data on intervention implementation, cost, equity and outcomes. The authors used PROGRESS-Plus to extract equity-relevant data from the studies and examined equity effects for each age group [21]
	Lay health workers in primary and community health care for maternal and child health and the management of infectious diseases [26]	This review included studies conducted in any country with any population as long as the intervention was delivered by lay health workers and intended to improve maternal and child health. Many of the included studies focused on low income populations and found that lay health workers can improve access to health care for low income groups and, if extrapolated to other settings, may contribute to reducing inequities [26]
	Built environment interventions for increasing physical activity in adults and children (Protocol) [27]	This review aims to examine the effectiveness of all built environment interventions to increase physical activity. If sufficient data are available, the authors plan to conduct subgroup analyses to explore whether there is likely to be a relationship of effect to disadvantage and whether an equity gradient is present by assessing studies that have included subgroup analyses by ethnicity, occupation, gender, education, socioeconomic status and disability (including individuals with specific morbidities) [27]

## Results

### Recommendations for assessing health equity

Health equity can be considered at the following ten steps in the systematic review process.

1. ***Define conceptual approach to health equity***

Systematic review authors should consider the relevance of health equity questions at the protocol stage by considering whether social gradients exist in the burden of the disease and whether relative or absolute effects of interventions are likely to differ for disadvantaged populations. When developing the protocol for a systematic review, it is important to define why there is a need to focus on health equity and the method of assessing disadvantage, including whether proxies will be accepted and, if so, which ones are most appropriate. For example, living in a rural village in a low- or middle-income country was accepted as a proxy of poverty and socioeconomic disadvantage for a review of school feeding [19].

2. ***Develop a theory-based approach, which may include an analytic framework that identifies health equity as an outcome***

**Figure 1:**
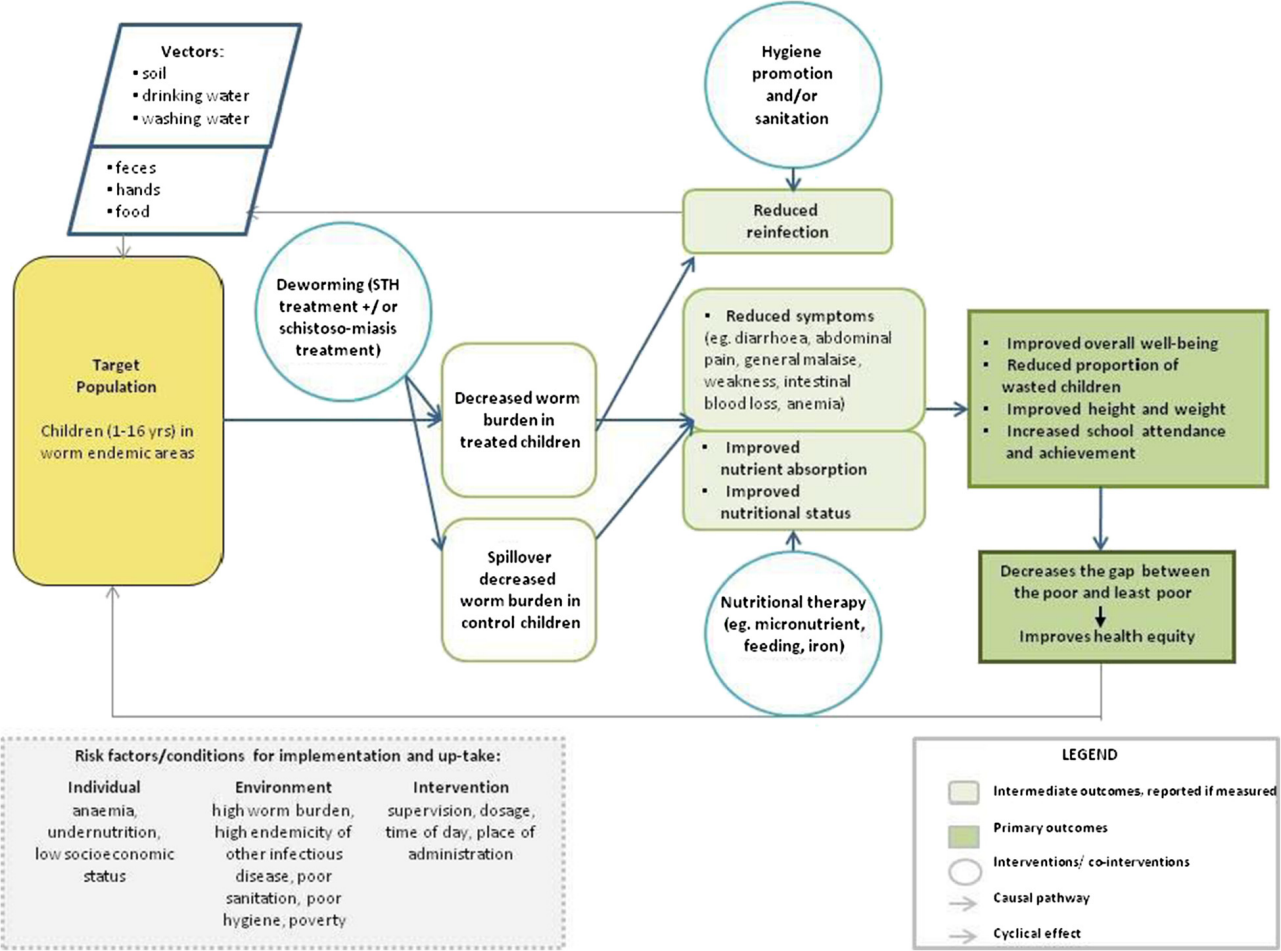
**Example analytic framework.** Source: Welch, 2013 [30].

In equity-focused reviews, it is important to define the assumptions and presumed causal pathways that will be assessed by the systematic review and how these are expected to affect health equity. Causal pathway analysis involves an assessment of contextual factors and processes that influence the effect of an intervention on health outcomes. For example, a systematic review of water and sanitation interventions assessed whether the hardware functioned properly to clean or filter the water, whether people used the hardware and finally the effects of diarrhea on health outcomes [28]. A visual representation (analytic framework) of the assumptions, causal pathways and likely effects on health equity may be useful to justify the equity questions, as well as identify important effect modifiers, confounding factors and important contextual factors [29]. An example analytic framework is provided in Figure 1, which shows how deworming of children is expected to improve health equity [30].

3. ***Frame the health equity questions***

Health equity questions must be defined across the Population, Intervention, Comparison, Outcome and Context or setting (PICO-C) if the review topic focuses on intervention effectiveness-related questions. Conceptualizing the review questions related to health equity requires a description of how the intervention is expected to work and why it may work differently depending on the context for disadvantaged populations or across gradients in socioeconomic status. This requires a consideration of both relative and absolute effects, as well as baseline risk of the health outcome of interest across social gradients. The absolute effect provides the difference in effectiveness between the most and least disadvantaged while the relative effect describes the difference in effectiveness relative to a reference group, such as the whole population [31]. Since disadvantaged populations may have worse health status and higher risk of adverse outcomes, interventions may have a greater absolute effect in disadvantaged populations, even if the relative effect is the same. For example, foreign-born Canadians have an incidence rate of tuberculosis that is 20 times higher than for non-Aboriginal Canadians (16 cases per 100,000 versus 0.8 cases per 100,000). Thus, in a Canadian guideline on tuberculosis for immigrants, although the relative effect of isoniazid preventive treatment was assumed to be 0.40 for both immigrants and Canadian-born, the difference in absolute risk means that the expected absolute benefit was 32 fewer cases of active tuberculosis per 1,000 people for immigrants compared to only 6 fewer per 1,000 for Canadian-born subjects [32].

4. ***Include relevant study designs to assess health equity questions***

Eligible study designs should be included and described according to their “fitness for purpose”, and the rationale should be clearly stated and explained [33]. For systematic reviews with a focus on health equity, the type of intervention (e.g., legislation applied at the population level) and the time frame of interest (e.g., long-term outcomes of interest not likely to be assessed in a short-term RCT) may require the inclusion of nonrandomized studies to inform the review. Upstream, policy-level interventions may have been evaluated in nonrandomized evaluations such as natural policy experiments (e.g., effects of privatization of public utilities, interventions to promote cycling and slum upgrading strategies) and thus necessitate the inclusion of a wider array of evidence [9,28,34]. For example, a systematic review that aimed to assess the health effects of complex housing improvement interventions included non-randomized study designs [35].

When equity is a main focus, the authors should consider additional study designs. A review of interventions to upgrade slums included both controlled before and after studies and interrupted time series as well as ‘supporting studies’ such as uncontrolled before and after studies and non-randomized, controlled studies with post-intervention outcome data [34]. Nonrandomized study designs provide considerations of the effects of context, setting and underlying mechanisms of action, which are important when evaluating a complex intervention, even if equity is not the main focus of the review. If the authors do not consider nonrandomized study designs, then failure to find assessment of effects on health equity may be due to “the inverse evidence law”, i.e., that there is less evidence available on the interventions that are most likely to influence policy and population health [36].

5. ***Identify information sources for health equity questions***

Searches related to disadvantage need to draw on social, political, cultural and ethical perspectives. Thus, potentially relevant studies may be found in a wide range of literature sources (such as books, government publications, policy documents and other gray literature), which are difficult to scope in terms of total volume, location and categorization. It is inappropriate and inaccurate to rely solely on conventional databases such as MedLine; topic-specific databases addressing the research topic such as transportation databases for questions about traffic calming could be more relevant than general databases [8,37].

6. ***Define search terms for health equity questions***

It is especially important to consider the risks of missing relevant literature when using filters for any concept, including disadvantage or health equity, as many of the words describing disadvantaged populations or settings are not indexed in the major databases. The use of text words to limit the search to concepts of health equity or disadvantage risks missing relevant studies that have been described using different terms (for example, disparities vs. inequities) [38]. Furthermore, some community-based interventions in low and middle income countries are entirely equity-focused by focusing on improving health outcomes for under-served populations, but are not indexed with any terms to describe health equity, disparities or inequalities. There are no validated health equity search filters, and equity terms are not indexed consistently [39]. Systematic reviewers need to plan for extra time to screen potentially relevant studies for health equity and should avoid using textword limits unless they have been validated, for example, in the Child Health filter [40].

7. ***Develop data extraction tools for health equity***

Data extraction tools should include specific fields for disadvantage and health equity, as well as any within-study assessment of the effect on health equity as an outcome. We recommend defining all factors of interest in a data extraction checklist to reduce the risk of missing important information. This may include proxy indicators for disadvantage, such as nutritional status. Use of the PROGRESS-Plus framework can ensure that this important information is captured.

8. ***Assess the influence of context and process on health equity outcomes***

This includes using methods to assess the influence of context and process on the effects of the intervention. This is most often done using standard systematic review methods, and the role of context can be explored using meta-analysis. Other review methods are also being increasingly applied to the exploration of context and process, such as realist evaluation [41], meta-ethnography [42] and thematic synthesis [43]. Guidelines for how to use these methods as part of a systematic review have been proposed by the Cochrane Qualitative and Implementation Methods Group (http://cqrmg.cochrane.org), the Cochrane Public Health Review Group [12] and others working in the area of theory-based systematic reviews [42]. The choice of method depends on a number of factors such as the types of questions posed, the types of data sources, and the outcomes and processes of interest. However, there is to date no comprehensive comparison of each of these methods, their advantages and disadvantages and how to choose one method over another, though work is underway to compare them [44]. The methods can also be used in tandem. For example, a review of school feeding used two methods to assess the role of process and context. A process evaluation tabulated effect sizes across implementation factors hypothesized to be important such as supervision and caloric content [19]. A realist evaluation was then used to propose policy recommendations about designing successful school feeding programs [45].

9. ***Use synthesis approaches to assess effects on health equity***

Questions about effects of interventions on health equity are likely to require additional synthesis approaches. These approaches may include meta-regression, subgroup analyses and sensitivity analyses, which are well described in the Cochrane Handbook [46] and other sources. As with any such analyses, these analyses need to be conducted according to existing quality standards such as *a priori* specification and use of other evidence to support hypotheses, such as other empiric evidence, within study effects supporting between-study differences and use of interaction tests [47]. Furthermore, these synthesis approaches may be used to test assumptions about the intervention using a causal pathway approach, which may strengthen inferences made based on these analyses. For example, a systematic review of HIV prevention interventions included an assessment of HIV causal pathways. The interventions were matched to HIV prevention goals along the proposed causal pathways to HIV infection [48].

10. *** Collect data related to applicability and equity***

Judgments about the applicability of findings to different settings and populations must be made by the user of a systematic review. However, systematic review authors can assist this decision-making by providing details about the settings and populations in the primary studies, as well as exploring the mediating effect of factors identified in the analytic framework. Second, the systematic review can provide an assessment of the applicability to the most likely setting and population, given the body of evidence. This assessment should present both relative effects and absolute effects, which may be crucially important for health equity questions. The transparent reporting of these factors and their mediating role is likely to be helpful for the end-user of the systematic review.

#### How to report the results of an equity-focused systematic review?

The Equity Methods Group has developed reporting guidelines specific to systematic reviews focusing on equity questions: PRISMA-E 2012 [15]. These guidelines were launched at the Second Global Symposium on Health Systems Research in Beijing, China. Additionally, an equity checklist for use in planning systematic reviews is available from the Campbell and Cochrane Equity Methods Group (http://equity.cochrane.org/).

#### Knowledge translation methods for an equity-focused systematic review

For these equity-focused reviews, the end result will not be an incontrovertible message as the evidence will often be suggestive given that the evidence base is underdeveloped and may also vary for populations and contexts. Therefore, knowledge translation methods need to assemble the best available evidence and help end-users to use it to make better decisions about how and where to intervene.

A multitude of frameworks for translating systematic review findings to policy and practice have been published [49-51]. The framework developed by Grimshaw et al. [52], drawing on Lavis et al. (2003) [53], suggests five questions that need to be addressed in developing a knowledge translation strategy. These are particularly pertinent for equity-focused systematic reviews given that they usually have more relevance to minority populations and/or developing country populations where decision-makers may not be aware of systematic reviews. The knowledge translation plan should be specific to the end-users, keeping in mind their awareness of systematic reviews.

(1) **What should be transferred?** Up-to-date systematic reviews or other syntheses of global evidence are useful for decision-makers who need to consider a range of equity-related issues (i.e., beyond those described in single studies). Products emanating from these reviews may include structured and/or tailored summaries, patient decision aids, clinical practice guidelines and policy briefs. Evidence products should include a consideration beyond “what works” to consider for whom interventions work (or not), why and at what cost.

(2) **To whom should research knowledge be transferred**? Equity-focused systematic reviews could be relevant to many different audiences including national/provincial policymakers in low- and middle-income countries, international aid agencies and practitioners.

(3) **By whom should research knowledge be transferred**? Building credibility as a messenger is an important consideration and requires a tailored approach [54]. Different messengers are needed depending on the nature of the message, especially in a field where the political dimension of the message is an issue to be considered.

(4) **How should research knowledge be transferred**? There is limited evidence, beyond the clinical context [52], about the effectiveness of knowledge translation strategies in general, let alone in reducing inequities. However, the literature suggests that any strategy is more likely to be successful if an assessment of the likely barriers and facilitators informs the choice of the specific interventions.

(5) **With what effect should research knowledge be transferred**? There is still controversy about what endpoints should be considered and how they should be measured [55]. Appropriate outcomes for evaluating a specific KT strategy should be selected, and they may vary across different stakeholder groups and occur at individual, organizational and system levels [55]. Disadvantaged groups may differ in the outcomes they value compared to the more affluent. The explicit use of evidence in the policymaking process (recognizing the range of other influential factors to be considered in the process) is a commonly used outcome [55,56].

The five questions above can be used as a general template by those designing, implementing and evaluating KT interventions. Although the evidence base to guide the choice of KT approaches targeted at policymakers is evolving [55,57], a profusion of innovative approaches exists that warrants further evaluation in the future.

Integrated knowledge translation implies that relevant knowledge users (practitioners, policymakers, patients and public) need to be involved in formulating the systematic review question and methods. For questions relevant to low- and middle-income countries (LMIC), who bear the highest burden of morbidity and mortality for all diseases, there is an urgent need to increase the representation of authors from LMIC in systematic review teams [58] because these authors can assist in actively translating/transferring and exchanging results with target audiences such as policymakers in LMICs. Initiatives by funders such as the WHO Alliance for Health Systems and Policy Research, the International Initiative for Impact Evaluation (3ie), Ausaid, DFID and CIDA are developing the capacity to conduct systematic reviews in low- and middle-income countries.

## Discussion

The 2015 deadline for the Millennium Development Goals (MDGs) is rapidly approaching, yet the 2010 Millennium Development Goals Report revealed that without a major push, many of the MDG targets are likely to be missed [59]. A major obstacle to the progress of the MDGs has been the inability of health systems in many low- and middle-income countries to effectively implement evidence-informed interventions. There are many examples of systematic reviews of high priority topics that can be used to inform policy-making to achieve the Millennium Development Goals. These include the use of zinc for the treatment of childhood diarrhea and of insecticide treated bednets to prevent malaria. However, the differences between the objectives of researchers and policymakers remain difficult to bridge. Equity informed reviews and their policy recommendations can help to bridge the knowledge translation gap by providing policymakers with synthesized evidence in a form that identifies effects in disadvantaged groups, thus aiding with the development and implementation of policies and programs aiming to meet priority health objectives [60].

In order to assist these objectives of improving the evidence base for health equity-focused policy questions, the Campbell and Cochrane Equity Methods Group has developed reporting guidelines specific for systematic reviews focusing on equity questions: PRISMA-E 2012 [15]. Additionally, guidance on conducting systematic reviews with a focus on health equity is in development to be added to the next major update of the Cochrane Handbook, and a health equity checklist for use in planning of systematic reviews is available online (http://equity.cochrane.org/).

## Conclusions

We hope that uptake and implementation of these recommendations will contribute to increased production and use of evidence on the effects of national and international policies and programs intended to take action on social determinants of health and reduce health inequities. Knowledge translation of these equity-focused systematic reviews that takes into account the context-dependent effects on health equity and focuses on appropriate knowledge users will contribute to increased awareness about the role of systematic reviews for equity-oriented decision-making.

### Summary points

•Systematic review authors should determine whether equity considerations are relevant for their review at the question formulation stage and then plan their review accordingly.

•This article proposes ten steps in the systematic review process where reviews can consider effects on health equity including framing the question, choosing methods, collecting data, and assessing the role of context and implementation methods.

•In order to maximize the effects of considering health equity in systematic reviews, knowledge translation steps are recommended that focus on the appropriate end-users and recognize that messages are likely to be suggestive and context-dependent.

## Competing interests

All authors have completed the ICJME unified disclosure form at http://www.icmje.org/coi_disclosure.pdf (available on request from the corresponding author); they have no financial relationships with any organizations that might have an interest in the submitted work. The corresponding author, MP, PT and JO are members of the Campbell and Cochrane Equity Methods Group, which has an interest in promoting the consideration of health equity in systematic reviews.

## Authors’ contributions

All authors contributed to the drafting and editing of the manuscript. PT, VW, MP, JO, EW and EK contributed to the chapter in development for the *Cochrane Handbook for Systematic Reviews of Interventions* on which the article is based. VW will act as guarantor. All authors read and approved the final manuscript.
